# Nursing Intervention Practices for Smoking Cessation: A Large Survey in Hong Kong

**DOI:** 10.3390/ijerph15051046

**Published:** 2018-05-22

**Authors:** Yim Wah Mak, Alice Yuen Loke, Frances K. Y. Wong

**Affiliations:** School of Nursing, the Hong Kong Polytechnic University, Hong Kong, China; alice.yuen.loke@polyu.edu.hk (A.Y.L.); frances.wong@polyu.edu.hk (F.K.Y.W.)

**Keywords:** smoking cessation, nursing intervention, 5 A’s

## Abstract

Previous studies have shown that nursing interventions are effective in helping people to stop smoking, but that the participation of nurses in tobacco control activities has been far from satisfactory. The primary objective of this study is to identify factors that encourage or discourage nurses from participating in providing smoking-cessation interventions to their clients, based on the 5 A’s (ask, advise, assess, assist, arrange) framework. A cross-sectional survey was conducted among 4413 nurses in Hong Kong from different clinical specialties. A logistics regression analysis found that predictors for the practicing of all of the 5 A’s are nurses who want to receive training in smoking-cessation interventions, those who have received such training, and those who are primarily working in a medical unit or in ambulatory/outpatient settings. The regression model also showed that attitude towards smoking cessation was positively associated with all of the 5 A’s. The results indicate a need to encourage and provide nurses with opportunities to receive training on smoking-cessation interventions. Strategies to persuade nurses to provide smoking-cessation interventions are also important, since nurses are motivated to perform smoking-cessation interventions when they feel a stronger sense of mission to control tobacco use.

## 1. Introduction 

The World Health Organization (WHO) has acknowledged that the tobacco epidemic is one of the biggest public health threats that the world has ever faced [1]. In response to this public health threat, different tobacco control strategies have been formulated. Although increasing the tax on tobacco is generally thought to be the most effective tobacco control measure, smoking-cessation programs are also a cost-effective way of helping people to quit smoking. These interventions are considered to be effective at reducing smoking-related mortality and morbidity, in comparison to passive health education. For example, pharmacotherapy and counseling have been proven to be effective at increasing quit rates [2,3,4,5,6]. Of the counseling approaches, nursing interventions for smoking cessation have been shown to be effective [2,7]. Nurses are the largest group of health providers and have many opportunities to work with people with health needs [8]. As most nurses are non-smokers, they are in a very good position to provide interventions to smokers [8,9]. However, studies have shown that the participation of nurses in tobacco control activities has been far from satisfactory [10,11].

Sufficient knowledge and active participation in tobacco control efforts can improve the effectiveness of such efforts and also enhance the quality of the nursing intervention that is being provided. Understanding the factors that affect the participation of nurses in smoking-cessation interventions may be of use in improving the nursing curriculum and in developing programs to educate both nurses and the public about the importance of controlling the use of tobacco. Although there is a similar study on the knowledge, attitudes, and practices (KAP) of nurses in China with regard to tobacco control [10], such a study has yet to be conducted in Hong Kong. China has ratified the Framework Convention on Tobacco Control (FCTC) of the WHO, the application of which has been extended to Hong Kong since 2006. Moreover, Hong Kong is considered a gateway to tobacco control efforts in mainland China. For example, nearly one third of the population in mainland China are smokers, with 52.9% of men and 2.4% of women considered active smokers [12]. Mainland Chinese consume nearly 40% the cigarettes produced in the world—more than those in the other top four tobacco-consuming countries put together [13]. In comparison, in Hong Kong the prevalence of smoking is only 10.0%, with 18.1% of men and 2.7% of women being smokers [14], and with the record being just 23.3% in 1982 [15]. The reason why the figures differ so greatly between Hong Kong and mainland China is not only because smoking-cessation programs began earlier in Hong Kong [11], but perhaps also because of the tobacco control attitudes and advocacy efforts of health care providers [16]. To further strengthen tobacco control efforts among the Chinese population and around the globe, it is important to carry out a comprehensive large-scale study of the knowledge and practices of nurses in smoking-cessation interventions in Hong Kong. 

### Aim of the Study

The aim of this study is to examine the involvement/practices of nurses in smoking-cessation interventions in their daily clinical practice in Hong Kong. The primary outcome of this study is an instrument based on the 5 A’s model on smoking-cessation interventions recommended in the Treating Tobacco Dependence Clinical Practice Guideline put forward by the U.S. Agency for Healthcare Research and Quality [17,18]. The 5 A’s model was originally developed in the U.S. for smoking interventions [17]. It is an evidence-based approach that is appropriate for application to a broad range of behaviors and health conditions. For example, it has been adopted for smoking cessation [19] and further to promote oral health [20]. The model helps to detect, assess, and manage smoking-related risk factors. This is also a model that can be used in primary health care to provide structure to the interactions between health professionals and patients [21,22]. 

The 5 A’s represent: (1) Ask about tobacco use; (2) Advise to quit; (3) Assess; (4) Assist; and (5) Arrange. As this 5 A’s model can be implemented by a single branch of the medical profession, it is generally believed to be a cost-effective model when implemented by health care professionals [23]. In this study, participation in smoking cessation is determined by which of the 5 A’s the nurses demonstrated when helping their patients or clients to quit smoking, and by what factors are associated with participation in each of the 5 A’s. 

The following are the specific objectives of this study:

1. to describe the knowledge and practices of nurses regarding smoking cessation;
2. to describe the smoking status and second-hand smoke exposure of nurses; and
3. to identify factors that determine the participation of nurses in smoking-cessation interventions for their clients.

## 2. Methods

### 2.1. Design

This is a cross-sectional survey study with convenience sampling.

### 2.2. Sample and Data Collection

In collaboration with the 14 nursing colleges of the Hong Kong Academy of Nursing (HKAN), the survey questionnaires were distributed to practicing nurses from different health care settings, including public and private hospitals. The questionnaire was distributed to the workplaces of the nurses from 4 May to December 2013 via (i) the central nursing division (CND) of all hospitals of the Hong Kong Hospital Authority, (ii) the CND or equivalent of the five private hospitals, (iii) before the 14 sessions of educational talks on tobacco control to nurses working at the Hospital Authority, Department of Health, and other health care institutions, and (iv) during key activities organized by the Hong Kong Academy of Nursing during the data collection period. Under the above comprehensive coverage, which had already included 25 out of 43 public hospitals in Hong Kong, the sampling frame was considered adequate enough to represent all practicing registered nurses in Hong Kong. The eligible participants were qualified registered nurses currently working in settings where they are able to work directly with people with health needs. The target sample size was 3000—10% of the total number of registered nurses in Hong Kong. The target of 3000 was set because enough funding was available to conduct such a large-scale study. Moreover, based on the rule of thumb that a sample size of 15 is required to estimate the parameters of an independent variable in a regression analysis, the target sample size is appropriate for this study. Estimating a response rate of 52% [24], we distributed about 5800 sets of questionnaires [25]. 

### 2.3. Ethical Approval

The survey protocol was approved by the Human Research Ethics Approval Committee of the Hong Kong Polytechnic University (Ethical Approval letter is attached). The study was conducted with the approval of the Hong Kong Hospital Authority and five private hospitals in Hong Kong .

### 2.4. Instruments

Throughout the quantitative survey, validated measures were adopted to explore the key constructs of knowledge on the harms of smoking and the benefits of quitting, attitudes towards smoking cessation, practices in smoking cessation, and so on. All measures were administered to nurses in written Chinese. The measures were: 

*Knowledge of the Harms of Smoking and the Benefits of Quitting*. A 15-item instrument describing the harms of smoking (9 items) and the benefits of quitting (6 items) was adopted. Sample items included: “Smoking delays the recovery of wounds” (the harms of smoking), and “Quitting smoking can reclaim years of life that might have been lost due to smoking” (the benefits of quitting). For each item, the responses were given using a 5-point Likert scale ranging from 1 (strongly disagree) to 5 (strongly agree), with a Cronbach’s alpha of internal consistency of .89 in this study.

*Attitudes towards Smoking Cessation*. A 10-item instrument evaluating attitudes towards tobacco control initiatives was adopted [10]. The following is a sample item: “Tobacco control is an important part of health advocacy in Hong Kong.” The responses were given using a 4-point Likert scale ranging from 1 (very unimportant) to 4 (very important), with a Cronbach’s alpha of internal consistency of 0.82 in this study.

*Practices in Smoking Cessation*. Five items were used to evaluate the participation of the nurses in the 5 A’s in the previous year (Fiore et al., 2000). The 5 A’s were: “Ask about the smoking status of service users (Ask), Advise smokers to quit smoking (Advise), Assess the readiness of smokers to quit (Assess), Assist smokers in quitting smoking (Assist), and Refer smokers to smoking-cessation services (Arrange).” For each item, the responses were given using a 4-point Likert scale ranging from 1 (Never) to 4 (Often), with a Cronbach’s alpha of internal consistency of 0.85 in this study.

### 2.5. Data Analysis

To identify factors promoting the participation of nurses in the 5 A’s, a bivariable analysis was conducted among the nurses’ 5 A’s and all other variables, including: (1) the nurses’ demographic information and training received in smoking-cessation interventions; (2) smoking-related variables, such as the nurses’ smoking status and susceptibility to smoking; (3) determinants of the nurses’ participation in smoking-cessation interventions; and (4) the nurses’ attitudes towards smoking cessation as well as their knowledge of smoking and quitting. In the first group of variables, only the nurses’ age group was included but not their exact age because asking the nurses to reveal their exact age could have caused them to feel embarrassed. When analyzing the age group data, the age groups were represented as 1 to 5 rather than as an age grouped categorical variable to demonstrate the linear relationship between age and the outcome variable. The fourth group of variables was analyzed, with the variables being collapsed into attitude and knowledge scores by adding up the responses. The significant predictors identified in the bivariable analysis were further examined using multivariable logistic regression models. The level of significance was set at 0.05 (not 0.10) to limit the number of variables to be considered in a regression analysis, so that a more focused regression model could be obtained. The outcomes of the 5 A’s were first transformed from the 4-point Likert scale into the binary format of 0 (never and rarely) and 1 (sometimes and often), and then regressed on the aforementioned significant predictors using the forward stepwise (Wald) variable selection method available in the SPSS logistic regression function. The variance inflation factor (VIF) of each regression model was checked to ensure that the models did not have the problem of multicollinearity. All of the confidence intervals were calculated using the online calculator provided by the University of California, San Francisco, Clinical and Translational Science Institute [25].

## 3. Results 

### 3.1. Profile of the Nurses

Among the total of 4723 returned questionnaires, 298 were completed by nurses who were not eligible to participate in the survey because they were neither currently practicing nurses nor registered nurses, and 12 incomplete questionnaires were discarded. In the end, a total of 4413 questionnaires were analyzed and included in this report. The number of nurses who responded to this study comprises more than 10% of registered nurses in Hong Kong. 

The demographics and work nature of the nurses who completed the questionnaire are shown in Table 1 and Table 2. The majority of the nurses were female (84.7%) and under the age of 50 (85.7%). The registered nurses were general nurses (88.1%), psychiatric nurses (7.1%), and nurse midwives (9.4%). Over half of the nurses had attained a bachelor’s degree (53.7%), and a quarter had attained a master’s degree (27.3%) in nursing. The majority of the nurses (95.2%) had not received any training in smoking-cessation interventions, and 27.2% expressed an interest in receiving such training [26]. 

### 3.2. Prevalence of Active Smoking and Second-Hand Smoke Exposure among Nurses 

Among the 4413 nurses, 98.2% reported that they never smoked. However, 65.9% reported that they have family members or close friends who smoke. Those who stated that their family members or close friends sometimes or often smoke in their presence amounted to 49.9%. As many as 71.1% of the nurses were “sometimes or often” exposed to second-hand smoke in their daily life. Forty percent reported that their family members or close friends have developed smoking-related diseases such as cancers of the mouth and throat, lungs, oesophagus, urinary bladder, stomach, and so on; or cardiac or respiratory diseases (Table 3).

### 3.3. Knowledge of the Health Hazards of Smoking and the Benefits of Quitting 

Table 4 shows the knowledge of the nurses regarding the health hazards of smoking and the benefits of quitting. A majority of the nurses (60.4–81.3%) know that smoking is hazardous to various aspects of health, such as delaying recovery from wounds, increasing one’s risk of developing peptic ulcer, causing poorer muscle strength, and bringing on early menopause. Well over 84% of the nurses agreed that quitting smoking could protect others against the hazards of exposure to second-hand and third-hand smoke [27]. However, only 60% of the nurses knew that quitting smoking can lead one to reclaim years of life that might have been lost due to smoking, and that the chance of developing coronary heart disease could be cut in half that of a smoker’s one year after quitting smoking. 

### 3.4. Nurses’ Attitudes Towards Smoking Cessation 

Table 5 shows that the majority of nurses agreed that tobacco control is an important health advocacy program in Hong Kong (97.0%) and that nurses should act as role models and should not smoke (93.0%). As high as 80% of nurses agreed with the tobacco control legislation and with the assertion that nurses should assume an important role in tobacco control. However, less than half of the nurses (40.4–44.2%) believed that they were familiar with the smoking-cessation services and resources available in Hong Kong, confident about helping smokers quit, and equipped with the knowledge and skills required to help smokers quit. 

## 4. Nurses’ Practice of the 5 A’s of Smoking Cessation: Ask, Advise, Assess, Assist, Arrange 

Nurses were asked about their smoking-cessation practices, as measured using 15 questions in the 5 A’s framework: Ask, Advise, Assess, Assist, and Arrange (Table 6). Over half of the nurses frequently (sometimes and often) asked (59.1%) and documented (52.1%) the smoking status of their service users. Although they frequently (64.9%) advised smokers to quit smoking, only 35.1% of them assessed the smoker’s readiness to quit smoking. About 13.5% to 38.9% have utilized various ways to assist smokers to quit smoking. Only 20.1% would make arrangements for smokers to attend smoking-cessation services, and 10.5% would follow up on the progress of the smokers in quitting smoking. 

## 5. Determinants of Nurses’ Participation in Smoking-Cessation Interventions

Table 7 shows the determinants of nurses’ participation in tobacco cessation interventions. 

Over 80% reported that the important factors were the benefits to health of quitting (92.5%), time availability (89.3%), the motivation of smokers to quit (88.8%), support by their work unit (86.3%), adequate knowledge (88.2%) or skills (86.7%) to help smokers quit, as well as having the confidence to help smokers quit (83.4%). 

## 6. Factors Associated with Self-Reported Performance in Terms of the 5 A’s

Table 8 shows the results of applying a multivariable logistics regression model to the nurses’ self-reported participation in smoking-cessation interventions, in terms of the 5 A’s. The Nagellaerke R^2^ of the five models ranged from 0.08–0.23, with the models considered to have a medium effect size, with the exception of the first A’s model (Ellis, 2010). The significant predictors that were found can be divided into four groups: (1) nurses’ demographic information and history of training in smoking-cessation interventions; (2) smoking-related variables; (3) determinants of nurses’ participation in smoking-cessation interventions; and (4) nurses’ attitude and knowledge scores on smoking cessation. 

Regarding the nurses’ demographic information and history of training in smoking-cessation interventions, the predictors were quite consistent among the 5 A’s. Nurses who wanted to receive training in smoking-cessation interventions (Ask, OR: 1.59, 95%CI: 1.36–1.86; Advise, OR: 1.67, 95%CI: 1.41–1.98; Assess, OR: 1.75, 95%CI: 1.46–2.10, Assist, OR: 1.62, 95%CI: 1.36–1.94; Arrange, OR: 1.91, 95%CI: 1.59–2.30) and those who received the training (Ask, OR: 2.84, 95%CI: 1.91–4.22; Advise, OR: 1.97, 95%CI: 1.28–3.01; Assess, OR: 2.84, 95%CI: 1.90–4.23, Assist, OR: 2.62, 95%CI: 1.87–3.65; Arrange, OR: 2.92, 95%CI: 2.07–4.12) were more likely to participate on the 5 A’s. This was also the case with the nurses who worked primarily in the Medicine Department (Ask, OR: 1.41, 95%CI: 1.19–1.67; Advise, OR: 1.98, 95%CI: 1.65–2.38; Assess, OR: 1.65, 95%CI: 1.36–2.00, Assist, OR: 1.46, 95%CI: 1.20–1.79; Arrange, OR: 2.51, 95%CI: 2.05–3.08) and the Ambulatory/Outpatient Department (Ask, OR: 1.28, 95%CI: 1.01–1.62; Advise, OR: 1.84, 95%CI: 1.42–2.39; Assess, OR: 1.39, 95%CI: 1.05–1.85, Assist, OR: 1.59, 95%CI: 1.22–2.07; Arrange, OR: 3.23, 95%CI: 2.50–4.18). The results of the factors of gender and age group were slightly inconsistent. Female nurses were only more likely to advise (OR: 1.51, 95%CI 1.23–1.86) and assist (OR: 1.68, 95%CI: 1.35–2.09). More mature nurses were more likely to participate in providing advice (OR: 1.11, 95%CI: 1.04–1.20) and assistance (OR: 1.22, 95%CI: 1.12–1.32), and in making arrangements (OR: 1.20, 95%CI: 1.10–1.31), but were less likely to participate in asking questions (OR: 0.84, 95%CI: 0.78–0.90). 

With regard to smoking-related variables, nurses who had family members who suffered from smoking-related diseases were more likely to participate in making assessments (OR: 1.19, 95%CI: 1.01–1.41), while those who had been exposed to second-hand smoke were more likely to participate in asking (OR: 1.20, 95%CI: 1.03–1.39), advising (OR:1.37, 95%CI: 1.17–1.60), and assisting (OR:1.23, 95%CI: 1.02–1.50). 

For the predictors related to determinants of nurses’ participation in smoking-cessation interventions, no significant predictor was associated with all of the 5 A’s. Among the significant predictors, “Smokers’ motivation to quit” (Advise, OR: 1.40, 95%CI: 1.07–1.83; Assess, OR: 1.74, 95%CI: 1.22–2.47, Assist, OR: 2.65, 95%CI: 1.82–3.86), “Health benefits of quitting smoking” (Ask, OR: 1.92, 95%CI: 1.45–2.53; Advise, OR: 2.36, 95%CI: 1.70–3.26; Assess, OR: 2.01, 95%CI: 1.24–3.28; Arrange, OR: 2.30, 95%CI: 1.36–3.88), and “Time availability” (Ask, OR: 1.49, 95%CI: 1.18–1.89; Arrange, OR: 1.56, 95%CI: 1.07–2.30) were positively associated with only some of the 5 A’s.

Attitude and knowledge scores were the key variables in this study. The regression model showed that the attitude score was positively associated with all of the 5 A’s (Ask, OR: 1.06, 95%CI: 1.02–1.08; Advise, OR: 1.08, 95%CI: 1.05–1.12; Assess, OR: 1.22, 95%CI: 1.17–1.26, Assist, OR: 1.37, 95%CI: 1.31–1.43; Arrange, OR: 1.27, 95%CI: 1.21–1.32), although the knowledge score was only associated with advise (OR:1.04, 95%CI: 1.02–1.06), assess (OR:1.04, 95%CI: 1.02–1.06), and arrange (OR:1.05, 95%CI: 1.02–1.07). 

## 7. Discussion

Nurses make up the largest number of health care professionals in Hong Kong (FHB, 2017). Smoking-cessation advice and/or counseling can appropriately and effectively be provided by nurses in clinical practice to help patients to quit smoking. This is the first large survey of nurses in Hong Kong to study the associations between the frequency with which nurses deliver each of the “5 A’s” (Ask, Advise, Assess, Assist, Arrange) and the demographics, training received in smoking-cessation programs, professional factors, and the knowledge and beliefs about tobacco cessation of nurses practicing in public and private hospitals and in non-governmental organizations. Hong Kong is the gateway to mainland China, where smoking is prevalent; thus, this survey is important as an example to China. The results of this study will be of use to other countries seeking to develop tobacco control strategies. The results of this survey can also serve as baseline data for making comparisons with the tobacco control activities of other nurses, which have been conducted by the Nursing Charter on Tobacco Control Steering Group [28]. 

The majority of the respondents agreed that tobacco control is an important health advocacy program in Hong Kong (97.0%) and that nurses should act as role models and should not smoke (93.0%). On the other hand, over 50% of them disagreed with the statements that they are equipped with the knowledge and skills to help smokers to quit smoking (59.4%), confident about being able to help smokers to quit (57.7%), and familiar with the smoking-cessation services and resources available in Hong Kong (55.8%). In comparing these results with those in the report from a national survey by Sarna et al. [29], who found that 82.7% of nurses feel that they lack the skills, 75.2% the knowledge, and 43.6% the confidence to participate in smoking-cessation efforts, we found that our respondents had a higher level of knowledge and skills but were less confident about participating in smoking-cessation interventions. This shows that the finding that many nurses are not yet well prepared to intervene in tobacco cessation with patients might be due to the inadequate contents on smoking cessation in most nursing curricula in Hong Kong. The findings from the survey of four Asian nations by Sarna et al. [30] indicate that information on various tobacco control intervention strategies, such as controlling tobacco use, cessation, reducing exposure to second-hand smoke, and promoting health, should be included as part of nursing education, starting with primary education and continuing with post-graduate education for nurses in various specialties. It has also been suggested that content relating to tobacco control, clinical tobacco cessation techniques, and competence in the delivery of the cessation interventions be included in the undergraduate nursing curriculum, and reinforced in graduate programs for nurse practitioners and clinical nurse specialists [31,32]. 

Referring back to the regression model developed in this study, the above suggestions are consistent with the finding that “training received in smoking-cessation interventions” is the predictor with the highest odds ratio. As wanting to receive training is also an important predictor, skills training for nurses should be made available since the evidence has indicated that training health professionals, including nurses, to provide smoking-cessation interventions leads significantly more of their clients to quit smoking [33]. The regression model showed that nurses who work primarily in the ambulatory/outpatient and medicine areas were more likely to be frequent participants in the 5 A’s. Perhaps priority should be given to setting up on-the-job training courses for these nurses so that they can become role models for other nurses. 

Referring to the model predictors related to attitudes and knowledge on smoking and quitting, the findings are completely consistent with the above discussion. It is natural to expect that nurses with a better attitude and more knowledge on smoking cessation will do more to help their patients to quit smoking. In addition to the attitudes and knowledge of nurses, which can be improved through appropriate training, the willingness of nurses to participate in the 5 A’s was also dependent on outside factors. These outside factors include the availability of time and the motivation of smokers to quit. This result indicates that the nurses perceived helping people to quit smoking as “extra tasks” that they were not expected to take on. If they had the time and opportunity to help patients who are willing to be helped, then they would be more willing to do so. This finding indicates that more work needs to be done to improve the tobacco control attitudes of nurses and to encourage them to more proactively provide tobacco control interventions to their clients. Towards this end, providing nurses with training in motivational interviewing may be useful [34,35]. 

As for the smoking-related variables, it is difficult to draw any concrete conclusions. Perhaps this is because 98.2% of nurses in Hong Kong never smoke, resulting in few related predictors. Among the limited predictors, “Family members suffering from smoking-related diseases” was only positively associated with “assess”, while “Exposed to second-hand smoke” was only positively associated with “ask”, “advise”, and “assist”. The findings on these two predictors indicate that more personal reasons for participating in tobacco control interventions are important to nurses. Further research is required to come to any conclusions in this area.

### Limitations

Although it would have been ideal if the results could have been weighted based on the population profile of nurses in Hong Kong, as is the practice in a typical public opinion program, this was not done in this study because the population profile of the nurses was not available. The study also did not take into account the cluster effect of the hospitals, because the questionnaire did not include a question on the name of the hospital in which the respondent was working. According to Wong et al. (2017) [36], a big dataset with too much information on the subjects may lead to privacy problems. This is because the subjects’ identity could be uncovered based on the research data records. Although differences due to different cultures relating to smoking cessation within each hospital could be missed, the name of the hospital in which a nurse works was not included in the questionnaire to ensure that the nurses would have no reservations about completing the questionnaire. 

## 8. Conclusions

This study clarified the associations between the frequency with which nurses deliver each of the “Five A’s” and the demographics, training received in smoking-cessation programs, professional factors, and the knowledge and beliefs about tobacco cessation of nurses working in public and private hospitals and in non-governmental organizations. The regression model developed in this study indicates a need to encourage nurses and provide them with opportunities to receive training in smoking-cessation interventions. The findings of the study also point to the importance of external factors, such as the availability of time and the motivation of smokers to quit. All of the above findings can serve as a baseline and an example for researchers in China to conduct their own studies on smoking-cessation interventions.

## Figures and Tables

**Table 1:** Demographics of the nurses (N = 4413).

Factors		n ^#^	(%)
**Gender**	Female	3678	84.7
	Male	666	15.3
**Age groups**	20–29	1169	27.3
	30–39	1245	29.1
	40–49	1255	29.3
	50 and older	609	14.3
**Marital status**	Single	1831	42.3
	Married/Co-habiting	2388	55.2
	Divorced/Separated/Widowed	107	2.5
**Category of nursing registration**	General	3743	88.1
	Psychiatric	303	7.1
	Midwifery	398	9.4
**Highest educational qualification**	Diploma/Higher Diploma	770	17.2
	Post-graduate Diploma	23	0.5
	Bachelor’s Degree	2283	53.7
	Master’s/Doctorate Degree	1175	27.7

^#^ The numbers may not add up to 4413 due to missing data.

**Table 2:** Types of work settings, position, primary specialty area, and training relating to smoking-cessation interventions (N = 4413).

Factors		n ^#^	(%)	95%CI
**Type of institution**	Government	277	6.3	5.6–7.1
	Hospital Authority	3421	77.8	76.5–79.0
	Academic institution	28	0.6	0.4–0.9
	Others	674	15.3	14.3–16.4
**Current work position**	Registered Nurse	2920	67.5	66.1–68.9
	Nursing Officer	335	7.7	7.0–8.6
	Advanced-practice nurse	497	11.5	10.6–12.5
	Nurse specialist	80	1.8	1.5–2.3
	Nurse consultant	29	0.7	0.4–1.0
	Ward manager /Departmental operations manager	209	4.8	4.2–5.5
	General manger (Nursing)	3	0.1	0.0–0.2
	Others	253	5.8	5.2–6.6
**Primary specialty area**	Medicine	890	20.8	19.6–22.0
	Surgery	601	14.0	13.0–15.1
	Ambulatory/Outpatient	413	9.6	8.8–10.6
	Obstetrics	381	8.9	8.1–9.8
	Mental health/Psychiatric/Addiction treatment	272	6.4	5.6–7.1
	Pediatrics	257	6.0	5.3–6.8
	Geriatrics	249	5.8	5.1–6.6
	Accident and Emergency	233	5.4	4.8–6.2
	Rehabilitation	145	3.4	2.9–4.0
	Home visiting nurse	136	3.2	2.7–3.7
	Gynecology	122	2.9	2.4–3.4
	Administration/Management	93	2.2	1.8–2.7
	Teaching	49	1.1	0.8–1.5
	Public health	41	1.0	0.7–1.3
	Residential care	8	0.2	0.1–0.4
	Occupational health	2	0.0	0–0.2
	Others	388	9.1	8.2–10.0
**Training**	Training received in smoking-cessation interventions	208	4.8	4.2–5.5
	Wanted to receive training in smoking-cessation interventions	1153	27.2	25.8–28.5

^#^ The numbers may not add up to 4413 due to missing data.

**Table 3:** Active smoking and second-hand smoke exposure of nurses (N = 4413).

Items		n ^#^	(%)	95%CI
Smoking Status	Never	4296	98.2	97.8–98.6
	Ever	77	1.8	1.4–2.2
Do you have any family members or close friends who smoke?	Yes	2898	65.9	64.5–67.3
	No	1497	34.1	32.7–35.5
If you have family members or close friends who smoke, do they smoke around you?	Never	312	11.2	10.0–12.4
	Rarely	1088	38.9	37.1–40.8
	Sometimes	1163	41.6	39.8–43.5
	Often	232	8.3	7.3–9.4
In general, are you exposed to second-hand smoke in your daily life?	Never	102	2.3	1.9–2.8
	Rarely	1158	26.5	25.2–27.9
	Sometimes	2385	54.7	53.2–56.2
	Often	717	16.4	15.3–17.6
Are any of your family members suffering from smoking-related diseases?	Yes	1751	40.0	38.6–41.5
	No	1511	34.5	33.1–36.0
	Don’t know	1114	25.5	24.2–26.8

^#^ The numbers may not add up to 4413 due to missing data.

**Table 4:** Nurses who correctly answered the questions related to the health hazards of smoking and the benefits of quitting (N = 4413) ^#^.

Items		n ^##^	(%)	95%CI
Health hazards of smoking	Long-term passive smokers suffer a higher risk of developing smoking-related diseases than active smokers	3557	81.3	80.1–82.4
	The breast milk of female smokers contains nicotine	3330	76.1	74.8–77.3
	Smoking delays recovery from wounds	3283	75.0	73.6–76.2
	Smoking can cause impotence, penile erection dysfunction, and premature baldness in male smokers	3261	74.5	73.7–76.3
	Third-hand smoke contains many toxic substances that persist on the surface of objects for weeks or months	3123	71.9	70.6–73.3
	Smoking can cause peptic ulcers	3077	70.3	68.9–71.7
	Smokers have poorer muscle strength, agility, and balance	2906	66.3	64.9–67.7
	Smoking can cause early menopause	2661	60.9	59.4–62.3
	Smoking is as addictive as taking heroin	2647	60.4	59.0–61.9
Benefits of quitting	Quitting smoking can protect others against the hazards of second-hand and third-hand smoke	3687	84.1	83.0–85.2
	Quitting smoking can reclaim years of life that might have been lost due to smoking	2664	60.8	59.3–62.2
	The chance of developing coronary heart disease is cut in half that of a smoker’s 1 year after quitting smoking	2643	60.2	58.8–61.7
	The risk of dying from lung cancer is about half that of a smoker 10 years after quitting smoking	2452	56.0	54.5–57.4
	The risk of developing coronary heart disease is similar to that of a non-smoker 15 years after quitting smoking	2298	52.5	51.0–54.0
	The risk of stroke is reduced to that of a non-smoker 5 to 15 years after quitting smoking	2278	52.0	50.5–53.5

^#^ The numbers may not add up to 4413 due to missing data; **^##^** represents the number of interviewed nurses who correctly answered the question.

**Table 5:** Nurses’ attitudes (agree) towards smoking cessation (N = 4413) ^#^.

Item	n ^##^	(%)	95%CI
Tobacco control is an important health advocacy program in Hong Kong	4243	97.0	96.5–97.5
Nurses should act as role models and should not smoke	4065	93.0	92.2–93.7
I agree with Hong Kong’s tobacco control legislation	3676	84.3	83.1–85.3
Nurses should assume an important role in tobacco control	3541	80.9	79.7–82.1
Addiction to smoking can be stopped completely	3501	80.0	78.8–81.2
I want to participate in smoking-cessation work	2884	65.9	64.5–67.3
I understand Hong Kong’s tobacco control legislation	2523	57.7	56.2–59.2
I am familiar with the smoking-cessation services and resources available in Hong Kong	1935	44.2	42.8–45.7
I am confident about helping smokers quit	1849	42.3	40.8–43.7
I am equipped with the knowledge and skills to help smokers quit	1772	40.6	39.1–42.0

^#^ The numbers may not add up to 4413 due to missing data; ^##^ represents the number of participants who agreed with the item.

**Table 6:** Nurses’ practice of the 5 A’s of smoking cessation: Ask, Advise, Assess, Assist, Arrange (N = 4413) ^#^.

*5 A’s of Smoking-Cessation Practices*	Never n (%) (95%CI)	Rarely n (%) (95%CI)	Sometimes n (%) (95%CI)	Often n (%) (95%CI)
**Ask** About the smoking status of service users	876 (19.9) (18.7–21.1)	925 (21.0) (19.8–22.2)	1191 (27.1) (25.7–28.4)	1410 (32.0) (30.7–33.4)
Document the smoking status of service users	1135 (25.8) (24.5–27.1)	974 (22.1) (20.9–23.4)	974 (22.1) (20.9–23.4)	1320 (30.0) (28.6–31.4)
**Advise** smokers to quit smoking	623 (14.2) (13.1–15.2)	925 (21.0) (19.8–22.3)	1732 (39.4) (37.9–40.8)	1121 (25.5) (24.2–26.8)
**Assess** the readiness of smokers to quit	1162 (26.5) (25.2–27.8)	1691 (38.5) (37.1–40.0)	1144 (26.1) (24.8–27.4)	393 (9.0) (8.1–9.8)
**Assist**				
Smokers in quitting smoking	1732 (39.3) (37.9–40.8)	1686 (38.3) (36.9–39.7)	768 (17.4) (16.3–18.6)	217 (4.9) (4.3–5.6)
Provide information about the harmful effects of smoking	1089 (24.7) (23.5–26.0)	1519 (34.5) (33.1–35.9)	1356 (30.8) (29.4–32.2)	439 (10.0) (9.1–10.9)
Provide information on second-hand smoke	1419 (32.2) (30.8–33.6)	1770 (40.2) (38.7–41.6)	945 (21.5) (20.2–22.7)	271 (6.2) (5.5–6.9)
Provide information about the benefits of quitting smoking	1159 (26.3) (25.0–27.6)	1534 (34.8) (33.4–36.2)	1297 (29.4) (28.1–30.8)	419 (9.5) (8.7–10.4)
Provide information about methods for quitting smoking	1323 (30.1) (28.7–31.4)	1652 (37.5) (36.1–39.0)	1117 (25.4) (24.1–26.7)	309 (7.0) (6.3–7.8)
Carry out smoking-cessation counseling with smokers	2204 (50.0) (48.6–51.5)	1583 (35.9) (34.5–37.4)	462 (10.5) (9.6–11.4)	155 (3.5) (3.0–4.1)
Advise smokers to use medication to quit smoking	2261 (51.3) (49.8–52.8)	1551 (35.2) (33.8–36.6)	467 (10.6) (9.7–11.5)	127 (2.9) (2.4–3.4)
Advise smokers to use a smoking-cessation hotline	1795 (40.8) (39.3–42.2)	1566 (35.6) (34.2–37.0)	799 (18.1) (17.0–19.3)	243 (5.5) (4.9–6.2)
Advise smokers to seek smoking-cessation counseling	1728 (39.3) (37.8–40.7)	1478 (33.6) (32.2–35.0)	889 (20.2) (19.0–21.4)	307 (7.0) (6.2–7.8)
**Arrange**				
For smokers to attend smoking-cessation services	2100 (47.8) (46.3–49.3)	1412 (32.1) (30.7–33.5)	629 (14.3) (13.3–15.4)	255 (5.8) (5.1–6.5)
Follow up on the progress of smokers in quitting smoking	2546 (61.6) (60.1–63.1)	1153 (27.9) (26.5–29.3)	322 (7.8) (7.0–8.6)	113 (2.7) (2.3–3.3)

**Table 7:** Determinants of nurses’ participation in smoking-cessation interventions (N = 4413) ^#^.

Items	n	(%)	95%CI
Motivation of smokers to quit	3905	88.8	87.8–89.7
Benefits of quitting to health	4070	92.5	91.7–93.3
My expected success rate for quitting smoking	3066	69.9	68.5–71.2
Whether I am equipped with the knowledge to help smokers quit	3877	88.2	87.2–89.1
Whether I am equipped with the skills to help smokers quit	3811	86.7	85.4–87.5
Whether I am confident in helping smokers quit	3643	83.4	82.2–84.5
Availability of time	3896	89.3	88.4–90.2
Whether carrying out smoking-cessation interventions is my job responsibility	3233	74.2	72.9–75.5
Whether I have received recognition and rewards for my smoking-cessation work	2447	56.1	54.6–57.6
Support from my work unit	3585	86.3	85.2–87.3

^#^ The numbers may not add up to 4413 due to missing data; ^##^ represents the number of participants who considered the item to be an important issue.

**Table 8:** Logistic regression model of the 5 A’s (N = 4413).

Predictors	Ask ^†^		Advise ^†^		Assess ^†^		Assist ^†^		Arrange ^†^	
	OR	95%CI	OR	95%CI	OR	95%CI	OR	95%CI	OR	95%CI
Demographics and training history										
Gender **^††^**			1.51 ***	1.23–1.86			1.68 ***	1.35–2.09		
Age Group **^†††^**	0.84 ***	0.78–0.90	1.11 **	1.04–1.20			1.22 ***	1.12–1.32	1.20 ***	1.10–1.31
Want to receive training ^#^	1.59 ***	1.36–1.86	1.67 ***	1.41–1.98	1.75 ***	1.46–2.10	1.62 ***	1.36–1.94	1.91 ***	1.59–2.30
Training received in smoking-cessation interventions ^#^	2.84 ***	1.91–4.22	1.97 **	1.28–3.01	2.84 ***	1.90–4.23	2.62 ***	1.87–3.65	2.92 ***	2.07–4.12
Primary area of work: Medicine ^#^	1.41 ***	1.19–1.67	1.98 ***	1.65–2.38	1.65 ***	1.36–2.00	1.46 ***	1.20–1.79	2.51 ***	2.05–3.08
Primary area of work: Ambulatory/Outpatient ^#^	1.28 *	1.01–1.62	1.84 ***	1.42–2.39	1.39 *	1.05–1.85	1.59 **	1.22–2.07	3.23 ***	2.50–4.18
Smoking-related variables										
Family members suffering from smoking-related diseases ^#^					1.19 *	1.01–1.41				
Exposed to second-hand smoke ^#^	1.20 *	1.03–1.39	1.37 ***	1.17–1.60			1.23 *	1.02–1.50		
Determinants of nurses’ participation in smoking-cessation interventions										
Smokers’ motivation to quit ^##^			1.40 *	1.07–1.83	1.74 **	1.22–2.47	2.65 ***	1.82–3.86		
Health benefits of quitting smoking ^##^	1.92 ***	1.45–2.53	2.36 ***	1.70–3.26	2.01 **	1.24–3.28			2.30 **	1.36–3.88
Time availability ^#^	1.49 **	1.18–1.89							1.56 *	1.07–2.30
Attitudes and Knowledge on smoking and quitting										
Attitude score (0–10)	1.06 **	1.02–1.08	1.08 ***	1.05–1.12	1.22 ***	1.17–1.26	1.37 ***	1.31–1.43	1.27 ***	1.21–1.32
Knowledge score (0–15)			1.04 ***	1.02–1.06	1.04 **	1.02–1.06			1.05 ***	1.02–1.07
Nagellaerke R^2^	0.08		0.13		0.17		0.21		0.23	
VIF	1.01–1.16		1.01–1.49		1.01–1.45		1.01–1.09		1.04–1.17	

^†^ 0: never and rarely (reference), 1: often and sometimes; ^††^ 0: Female (reference), 1: Male; ^†††^ 1: 20–29, 2: 30–39, 3: 40–49, 4: 50 or above; ^#^ 0: No (reference), 1: Yes; ^##^ 0: unimportant (reference), 1: important; * *p* < 0.05; ** *p* < 0.01; *** *p* < 0.001.
